# Automated Lung Cancer Diagnosis Applying Butterworth Filtering, Bi-Level Feature Extraction, and Sparce Convolutional Neural Network to Luna 16 CT Images

**DOI:** 10.3390/jimaging10070168

**Published:** 2024-07-15

**Authors:** Nasr Y. Gharaibeh, Roberto De Fazio, Bassam Al-Naami, Abdel-Razzak Al-Hinnawi, Paolo Visconti

**Affiliations:** 1 Department of Electrical Engineering, Al-Balqa Applied University, Salt 21163, Jordan; nas@bau.edu.jo; 2 Department of Innovation Engineering, University of Salento, 73100 Lecce, Italy; roberto.defazio@unisalento.it; 3 Department of Biomedical Engineering, Faculty of Engineering, The Hashemite University, Zarqa 13133, Jordan; b.naami@hu.edu.jo; 4 Department of Medical Imaging, Faculty of Allied Medical Sciences, Isra University, Amman 11622, Jordan; abedalrazak.henawai@iu.edu.jo

**Keywords:** lung cancer, AI, Butterworth smooth filter, Chaotic Crow Search Algorithm and Random Forest (CCSA-RF), Multi-space Image Reconstruction (MIR) with Grey Level Co-occurrence Matrix (GLCM), Sparse Convolutional Neural Network (SCNN)

## Abstract

Accurate prognosis and diagnosis are crucial for selecting and planning lung cancer treatments. As a result of the rapid development of medical imaging technology, the use of computed tomography (CT) scans in pathology is becoming standard practice. An intricate interplay of requirements and obstacles characterizes computer-assisted diagnosis, which relies on the precise and effective analysis of pathology images. In recent years, pathology image analysis tasks such as tumor region identification, prognosis prediction, tumor microenvironment characterization, and metastasis detection have witnessed the considerable potential of artificial intelligence, especially deep learning techniques. In this context, an artificial intelligence (AI)-based methodology for lung cancer diagnosis is proposed in this research work. As a first processing step, filtering using the Butterworth smooth filter algorithm was applied to the input images from the LUNA 16 lung cancer dataset to remove noise without significantly degrading the image quality. Next, we performed the bi-level feature selection step using the Chaotic Crow Search Algorithm and Random Forest (CCSA-RF) approach to select features such as diameter, margin, spiculation, lobulation, subtlety, and malignancy. Next, the Feature Extraction step was performed using the Multi-space Image Reconstruction (MIR) method with Grey Level Co-occurrence Matrix (GLCM). Next, the Lung Tumor Severity Classification (LTSC) was implemented by using the Sparse Convolutional Neural Network (SCNN) approach with a Probabilistic Neural Network (PNN). The developed method can detect benign, normal, and malignant lung cancer images using the PNN algorithm, which reduces complexity and efficiently provides classification results. Performance parameters, namely accuracy, precision, F-score, sensitivity, and specificity, were determined to evaluate the effectiveness of the implemented hybrid method and compare it with other solutions already present in the literature.

## 1. Introduction

Lung cancer is responsible for the highest percentage of deaths that fall into the category of cancer-related illnesses worldwide. Since most people diagnosed with lung cancer are in an advanced stage of the disease, the prognosis for these patients is unfortunately not long-term [1,2]. Choosing the most appropriate therapy for lung cancer can be difficult for doctors due to several factors, including the advanced stage at which the disease is diagnosed, heterogeneity of imaging findings, and the histological tests in the diseased area [3]. Since the imaging features of lung cancer may vary from a single microscopic nodule to ground-glass opacity, several nodules or pleural effusion, collapsed lungs, and numerous opaque areas, it is difficult to correctly diagnose simple, tiny lesions [4]. Histo-pathological characteristics include adenocarcinoma, squamous or small cell carcinoma, and many other uncommon histological manifestations. Furthermore, the histology subgroups differ much further than that. For example, the classification of lung tumors published by the World Health Organisation in 2021 included a very large number of adenocarcinoma sub-types [5]. It is important to note that the clinical stage, histology, and genetic features of lung cancer all have a crucial impact in deciding the therapy options that are accessible [5].

With the evolution of precision medicine, physicians must gather all the relevant information before deciding whether to deliver chemotherapy, targeted therapy, immunotherapy, or maybe a combination of these treatments with surgery or radiation. When it comes to daily practice, the issue of whether or not to treat a specific condition is always a question that arises [6]. It would be helpful for clinicians to understand better the direct connection between the observations, interventions (inputs), and outcomes (outputs), thus determining a model that can be used for detecting, categorizing, or predicting diseases [7]. Currently, this information is derived from clinical studies and the expertise of medical professionals, causing physicians to become exhausted as they continually look through photos and/or pathology slides to arrive at a correct diagnosis. Reviewing patient documents to identify the most effective treatment alternatives also takes up a significant amount of time. The relevance of illness categorization and prediction may be shown by looking at information from previous years. The essential characteristics provided in a dataset must be well-known to determine the precise etiology of the illness in addition to the symptoms of the sickness [8]. Understanding the entire procedure would be much easier if there was a reliable prediction and categorization model.

The concept of artificial intelligence (AI) is introduced in this context. AI is a general term that includes a variety of algorithms that make predictions or classify objects based on data gathered in the past. AI has shown very promising results in terms of categorization and support in decision-making [8,9]. Machine learning (ML), a subfield of artificial intelligence, has sped up a lot of research regarding the medical profession [10,11]. On the other hand, Deep Learning (DL) is a subset of machine learning that focuses on Neural Networks (NNs) inspired by the human brain’s structure and function; it can analyze the specific aspects necessary for illness identification [12].

In the research conducted from 2014 to today, several apps and algorithms have been developed to support the medical profession by providing reliable results for patients. Machine learning has been the main impetus behind the rapid technological development in several fields, including natural language processing, automated speech recognition, and computer vision. These technologies have made it possible to design reliable systems, such as self-driving cars, automatic translation, and various other services. However, machine learning in medical care applications presents significant challenges despite all the progress made [13]. A considerable number of these problems were brought to light by medical care, which stated that the objective is to make accurate predictions, using the data gathered and controlled by the medical system [14]. AI algorithms analyze a given dataset using various methods to extract or highlight features from huge amounts of data; however, identifying an optimal arrangement of essential features and eliminating the repeating ones might be challenging. Considering such characteristics is complicated, and the measurements used to determine the accuracy are often imprecise [15]. Choosing a limited subset from a comprehensive range of characteristics is necessary to improve the model’s effectiveness. Following this, the dimensionality of the information will decrease due to the elimination of inconvenient and repetitive features, which will speed up the trained model in a manner comparable to boosting [16].

Principal Component Analysis (PCA) and Linear Discriminant Analysis (LDA) are two examples of practical methodologies used to extract relevant features from the current data [17]. In particular, selecting a feature has two essential goals in direct opposition to each other: first, to increase the presentation of the arrangement, and second, to reduce the number of features to overcome the dimensionality problem. Consequently, feature selection is vital for the above objectives [18]. In the next step, the feature enhancement approach was improved using choice-based multi-target techniques. Therefore, the methods utilized to choose efficient characteristics are the subject of this research study. Multiple strategies of image segmentation, feature selection, and regression utilizing Root Mean Square Error (RMSE) were used to identify tumor disease. The factors included recognizing patterns, finding objects, and categorizing the images. However, the methods that use the machine and deep learning models are constantly being upgraded. Therefore, it is problematic for researchers to discover an appropriate approach for evaluating the photographs and the strategies for selecting features that differ depending on the method used [19,20]. In addition, the classification accuracy is slightly reduced if the lung nodules are considered. To overcome these problems, this research work proposes effective solutions for all the above issues.

The lung cancer prediction using computed tomography (CT) images was performed using deep learning methods. However, the limits of the existing works cause the results to be poor, with inaccuracies during the detection. Some of the problems are listed below:

- Lack of image quality and resolution: the existing models are significantly influenced by the quality and resolution of the CT scans. Low-quality scans or those with artifacts introduce some noise, thus reducing the model’s effectiveness;
- Feature selection and algorithmic impact: feature selection accuracy depends on the algorithm’s choice and selected features’ number. Assumptions about critical characteristics for lung cancer detection may not always align with the model’s accuracy;
- Low-resolution image generation in the biomedical field: the existing image generation techniques face challenges such as less accuracy and high processing time while struggling with generating high-resolution images essential for the biomedical field;
- Sensitivity and model evaluation: in existing methods, the sensitivity fluctuations at low and high false positive (FP) rates, coupled with the impact of false negative rates on the F-1 score and negative predictive value (NPV), present challenges in achieving robust model evaluation and high performance.

This study attempts to solve the above concerns by applying a smoothening step to reduce the noise impact, followed by investigating the effect of utilizing the dual Chaotic Crow Search Algorithm and Random Forest (CCSA-RF) and Multi-space Image Reconstruction with Grey Level Co-occurrence Matrix (MIR-GLCM) to classify the features of interest (i.e., avoid unnecessary features) automatically, and followed with the employment of Sparse Convolutional Neural Network with a Probabilistic Neural Network (SCNN-PNN) to finally remove the redundant features and reduce complexity in the classification process. The carried-out experiments showed profitable accuracy in comparison to many similar studies. Therefore, in comparison to the literature, the main contributions of the proposed research are:

- A Butterworth smooth filter-based pre-processing can be applied to reduce the noise, that could be misinterpreted as features, before utilizing the CNN-based module. The efficacy of CNN models applied to smoothed images is investigated; this methodology provides good results in comparison to literature in which many attempts to segment lesions or increase the lesion quality, but this coincides with keeping noise that impacts the CNN models’ performance (i.e., accuracy) [21,22,23];
- To achieve better feature selection and extraction results, dual CCSA-RF and MIR-GLCM-based methods are performed to select the proper features, thus providing profitable outcomes for the lung-cancer CT image classification;
- To improve the classification accuracy, the Sparse Convolutional Neural Network with a Probabilistic Neural Network (SCNN-PNN)-based classification is applied to remove the redundant features from the attention module and provide high classification accuracy with less complexity.

The performance of the proposed work is evaluated based on several performance metrics, such as the F1-Score, accuracy, precision, sensitivity, and specificity.

The manuscript is arranged as follows: the next section illustrates the literature review, followed by the problem introduction and description of the proposed lung cancer detection framework (Section 2). Section 3 reports the experimental investigations to assess the performance of the proposed lung tumor detection method. An explanation of the experimental findings obtained from the suggested strategy is reported in Section 4. Finally, the approach and the potential future applications of this study are discussed in Section 5.

### Literature Survey

ML and DL techniques have been increasingly utilized in the medical field, particularly for image analysis and diagnostics tasks. In the case of lung cancer detection, these technologies offer promising avenues for faster and more accurate screening [24].

In [25], the authors presented a four-stage lung nodule detection model consisting of image pre-processing, segmentation, feature extraction, and classifying. Preprocessing is the first stage, during which the input picture goes through several actions. Next, the preprocessed images are segmented using Otsu’s thresholding model. The LBP features are then obtained in the third step and categorized using an improved Convolutional Neural Network (CNN). In this case, a suggested approach called Improved Moth Flame Optimization (IMFO) is used to optimize CNN’s activation function and convolutional layer count. Finally, the scheme improvement is verified using specific metrics. The main limitation of this approach lies in the reduced processing speed. In the future, the authors will apply different visualization tools to understand the elements collected by the networks for categorization and determine whether these features match those used by radiologists to diagnose patients. Radiologists also hope to apply the suggested technique to other lung pathologies.

In [26], the two components of the developed lung cancer diagnosis system are the segmentation, on top of the UNEt TRansformers (UNETR) network, and the classification part, developed on top of the self-supervised network, to classify the segmentation output as benign or malignant. Utilizing 3D-input CT scan data, the suggested approach offers a potent tool for early identification and management of lung cancer. Several tests have been conducted to enhance segmentation and classification results using the Decathlon dataset to test and train the model. The main limitations of the suggested model are its high computational demands and the need for a Graphic Processing Unit (GPU) to reach high performance. 

A. Bhattacharjee et al., in Ref. [27], presented an enhanced computer-aided diagnostic model based on deep neural networks called Xception. It comprises automatic multi-class image classification for computed tomography of the kidneys and lungs utilizing a fine-tuned network and transfer learning-based images net weights of the Xception framework. Despite promising results, the limitation of this approach was that the lack of training data prevented the suggested Malignant, Normal, and Benign (MNoB) model from outperforming the Normal, Cyst, Tumor, and Stone (NCTS) model. Expanding the training data by using data augmentation approaches may address this problem. Additionally, the two-dimensional nature of the proposed MNoB and NCTS models prevents them from extracting context from neighboring slices. A 3D classification model that can benefit from inter-slice context and improve model performance may be included to address this issue.

The authors in Ref. [28] presented two models based on MobileNetV2 and UNET to create an enhanced hybrid Neural Network (NN) for the semantic segmentation of dangerous lung tumors using CT images. The pre-trained MobileNetV2 was used as an encoder of a traditional UNET model for feature extraction, and the transfer learning approach was applied. The suggested network is a productive segmentation method using pointwise convolution to provide more characteristics and lightweight filtering to decrease the computation. Skip links were established between the UNET decoder levels and MobileNetv2 encoder layers employing the Relu activation function to improve the model convergence. This solution allowed the feature mappings generated by the encoder to be concatenated to the decoder at different resolutions. However, the research activity’s limitations should be considered; the suggested segmentation approach was tested only on the challenge’s validation set. It would need to be tested on different medical picture segmentation tasks, in addition to the challenge dataset, to determine its resilience. Although the segmentation results have not been fully processed, investigating the use of Conditional Random Fields (CRF) can potentially improve the segmentation precision. Furthermore, the model’s performance could be affected by its susceptibility to overfitting, especially when the training data are lacking or unbalanced. Adding more data during the training can reduce these concerns and prevent training data from being overstored.

Also, the authors in [29] proposed a 3D fast Region Convolutional Neural Network (R-CNN) attention-embedded for lung nodule detection. By giving the residual blocks more significant consideration and using Depth-wise Separable Convolution (DSC) instead of the 3D convolution of the first residual blocks, the ResNet is enhanced, which acts as the main feature-extraction network, shifting its focus to the main channels and regions of interest. Furthermore, it directs the high-level abstract semantic features and low-level location, contour, edge, texture, and shape characteristics to cooperate to generate complementary feature flows that extract modular features more accurately. DSC ensures sensitivity and accuracy of the nodule detection while reducing the model parameters and computations; it also improves the detection performance. However, the algorithm still has some drawbacks; the detection accuracy has increased but is still below the target level, and a low false-positive rate does not indicate a high sensitivity. Although focal loss can reduce the effects of information imbalance, it cannot completely eliminate them. Even when appropriate steps are taken to improve the data, a larger volume of data is needed as the network becomes deeper to extend the model’s capabilities.

Furthermore, the authors in [30] presented a deep neural model and a cloud-based data-collection technique for identifying the stages of pulmonary sickness, as well as a method for verifying and classifying distinct stages of lung tumor growth. The presented approach offers a hybrid method for positron emission tomography/computed tomography (PET/CT) images called Cloud-based “Lung Tumor Detector and Stage Classifier (Cloud-LTDSC)”. The Multi-layer Convolutional Neural Network (M-CNN) has been built and verified using standard benchmark pictures to identify various stages of lung cancer. However, the cloud system demonstrated memory constraints, preventing its use for analyzing more than 65 users’ records at once, making it inefficient for major hospital structures. In [31], S. Mithun et al. introduced multiple approaches for classifying radiological reports on lung cancer using rules-based and ML algorithms, such as XGBoost and DL algorithms based on bidirectional long short-term memory (LSTM) neural networks. For research and evaluation, 1700 radiological reports, including Computed tomography (CT) and PET/CT, were used. A drawback is that the entire pipeline is customized to extract diagnostic data only from the Health Information System (HIS); however, it could be adapted to extract data from other institutions. In addition, the rule-based model’s ontology was also tailored using just their data. After mapping most disease diagnostic terms, some false negatives remained unidentified in the lexicon. The models were trained to recognize reports with any of the three ideas or none since individual concept identification was impossible due to insufficient data in the concept classes. This limitation does not affect the rule-based scripts. The investigation had no negative detections. The dataset has no negative mentions of phrases. Thus, they must delete lung cancer references related to lobe, laterality, lesion description, etc. Now, they map ideas using the NCIT lexicon. SNOMED CT, ROO, and Radiology Lexicon may help with concept extractions, but they haven’t looked at them.

In [32], the authors developed two CNNs, including AlexNet and GoogleNet models, for lung tumor detection, using the LIDC datasets to pre-train CNNs. Pre-processed cancer images are used to train the CNNS to identify the specific lung regions suffering from the cancer. For classification purposes, these networks are outfitted with stratified architecture. The test results indicated that AlexNet and GoogleNet produce similar outcomes when considering time, initial learning rate, and accuracy. Table 1 summarizes the analyzed scientific works, highlighting the relative research gap.

This research work aims to investigate a new CNN-based approach applied to CT images for lung-cancer detection, utilizing the combination of a bi-level CCSA-RF algorithm for feature selection, MIR-GLCM for feature extraction, and SCNN-PNN-based classifier for removing the redundant features. These three procedures are applied to the CT images after the Butterworth high-pass filter (BHPF). The research objective is to investigate the operation of these three artificial intelligence procedures, which have not been tested before in the literature for CT lung-cancer automated diagnosis, attempting to reduce the CNN complexity while maintaining high classification accuracy. 

## 2. Materials and Methods

Firstly, this section introduces the main limitations of the solutions reported in the scientific literature to detect and classify lung tumors from CT images; then, the solutions proposed to take a step forward to improve the models’ performance are reported. Afterward, the architecture of the proposed model for lung tumor detection is presented.

### 2.1. Problem Statement and Research Solutions

This section discusses some significant solutions presented in the literature for detecting lung tumors from CT images, highlighting their main issues and limitations; based on this analysis, the proposed solution is presented to overcome the current limitations.

In [34], the authors proposed a new Deep Learning (DL)- based technique to diagnose lung cancer disease by extending layering to the original DenseNet framework and altering the DenseNet201 model. The proposed algorithm, trained using the Kaggle chest CT-scan image dataset, classifies lung cancer into 4 types: big cell tumors, normal cells, adenocarcinoma, and squamous cell carcinoma. Two feature selection approaches were utilized to choose the best features retrieved by DenseNet201. These features were then used for different ML classifiers. The main limitations of this approach are as follows:

- The quality and resolution of CT scans can considerably influence the model’s performance. Scans that are low quality or contain artifacts may cause noise in the model, limiting its usefulness;
- It should be noted that the assumption that the chosen parameters are vital for lung cancer diagnosis is not always correct. Additional features or variables can help identify lung cancer but are not included in the model;
- The method used and the number of features chosen can affect the accuracy of the feature selection approach.

Furthermore, in [35], the authors used multi-space images in the pooling layer of a CNN to increase the overall prediction accuracy while reducing the processing time. The proposed method optimizes lung cancer detection using Adam’s algorithm to optimize multi-space images in the pooling layer of the CNN with an auto-encoder system. Initially, convolution filtering was used to pre-process the CT images, and then max-pooling was used to down-sample the images. The autoencoder model has been subsequently utilized to extract features based on a CNN. Then, the multi-space image reconstruction technique is used to reduce errors during image reconstruction and increase the accuracy of lung nodule prediction. The reconstructed images are finally sent to the SoftMax classifier to classify the CT images. The main problems lie in the limitations introduced by the pooling layer of the neural network. The Reconstructed Image Subspace Analysis (RISA) was employed for image reconstruction featured low accuracy and long processing time. Large-window filters were unable to recreate images effectively using the RISA network, which affected classification accuracy and resulted in a long processing time. Furthermore, in [36], Z. Ren et al. presented a Lung Cancer Data Augmented Ensemble (LCDAE) architecture to address overfitting and poor performance issues in the classification tasks related to lung cancer. The LCDAE is made up of 3 elements: the Deep Convolutional GAN capable of synthesizing lung cancer images, and a Data Augmented Ensemble model (DA-ENM) that used a lung cancer dataset to train, test, and validate six refined transfer learning models. The data augmentation methods in the LCDAE are then combined in the third section, called Hybrid Data Augmentation (HDA).

The main issues are given below:

- The Lung Cancer Deep Convolutional generative adversarial networks (LDCGAN) are unable to produce high-resolution images, crucial for the biomedical field;
- The ensemble model requires a lot of time and computing resources to train.

In [37], the authors introduced a hybrid framework known as Lung cancer generative adversarial networks together with transfer learning (LCGANT) for lung cancer classification; it comprises two main components, the first is a deep convolutional Generative Adversarial Network (LCGAN) to create synthetic lung cancer images. The second component is a regularization-enhanced transfer learning (Visual Geometry Group VGG-DF) model to classify lung cancer images into three groups. The test results demonstrated that the developed hybrid framework performed excellently in classifying lung cancer (i.e., 99.84% ± 0.156% accuracy, 99.84% ± 0.156% precision, 99.84% ± 0.156% sensitivity, and 99.85 ± 0.156% F1-score). Nevertheless, the synthetic images produced by LCGAN differ slightly from real photos. The generator provides photos with 64×64 resolution, which is insufficient for the biological and medical fields that require high-resolution images.

Furthermore, E. A. Siddiqui et al., in [38], utilized Gabor filters with an improved Deep Belief Network (E-DBN) and different classification algorithms in the proposed lung cancer classification approach. They employed two cascaded RBMs in the E-DBN, namely Gaussian-Bernoulli (GB) and Bernoulli-Bernoulli (BB). A Support Vector Machine (SVM) algorithm performs best for any approach. The suggested model classifies lung CT images using an SVM and E-DBN classifier to increase the performance. However, a decrease in sensitivity at low FP rates often corresponds with increased sensitivity at high FP rates. The model’s NPV is influenced by the F-1 score, which decreases as FN rates increase.

To solve these problems, the LUNA 16 dataset, a subset of the Lung Database Consortium (LIDC) and Image Database Resource Initiative (IDRI), was employed in this research work. The LUNA16 dataset consists of 888 CT scans, all acquired using a slice thickness of less than 2.5 mm. The dataset was reviewed by 4 expert radiologists who amended the clinical annotations on each CT scan, showing the location coordinates (X, Y, Z) of the presence of lung lesions and the corresponding diameter in mm. Then, all nodules with a diameter greater than 3 cm were validated by the agreement of 3 out of 4 specialists. Also, the detected lesions located less than 5 mm apart are merged into a single element. To facilitate the advancement of the nodule detection algorithm, we also employed lung segmentation images already processed by an automatic segmentation algorithm [39]. The LUNA16 dataset is arranged into 10 sections used for the 10-fold cross-validation. The dataset collection and annotation process are fully explained in [40,41].

A 3D region of interest was identified by extracting the entire lung nodule’s volume, and a smooth low-pass Butterworth filter was applied to effectively eliminate high-frequency noise. Because it functions in the frequency domain, the filter matrix size does not limit it. We focus on key criteria such as diameter, margin, spiculation, lobulation, subtlety, and malignancy to correctly detect lung cancer. Sparse Convolutional Neural Network with Probabilistic Neural Network detects lung cancer using effective characteristics. In medical image analysis, an integrated strategy using the Chaotic Crow Search Algorithm and Random Forest (CCSA-RF) is proposed to select the critical features such as diameter, margin, and malignancy. Multi-space Image Reconstruction (MIR) using Grey Level Co-occurrence Matrix (GLCM) was used to improve image analysis in feature extraction, enhance accuracy, and accelerate the processing time. Indeed, MIR’s sharp reconstruction integrates images from different sections to improve cancer categorization, while increasing the filter’s window size speeds up the data processing.

Furthermore, the SCNN allows the reconstruction of a high-resolution CT image by combining the overlapping patches. The loss function between the reconstructed and real high-resolution images improves the end-to-end variables of the neural network. The Probabilistic Neural Network requires little computing power and training time. To successfully diagnose lung cancer, the Hybrid Sparse Convolutional Neural Network with Probabilistic Neural Network (SCNN-PNN) can be used. A hybrid method for lung cancer detection combines Sparse Convolutional Neural Network to create high-resolution CT and average overlapping patches, with PNN for accurate detection. The SCNN uses the balanced accuracy or F1-Score to verify accuracy against balanced data. Calculating the arithmetic mean on class-retracting accuracy provides a balanced measure of accuracy in both positive and negative cases. In conclusion, PNN can correctly and effectively identify tumors, thus making the hybrid SCNN-PNN a suitable technique.

### 2.2. Proposed Work

**Figure 1:**
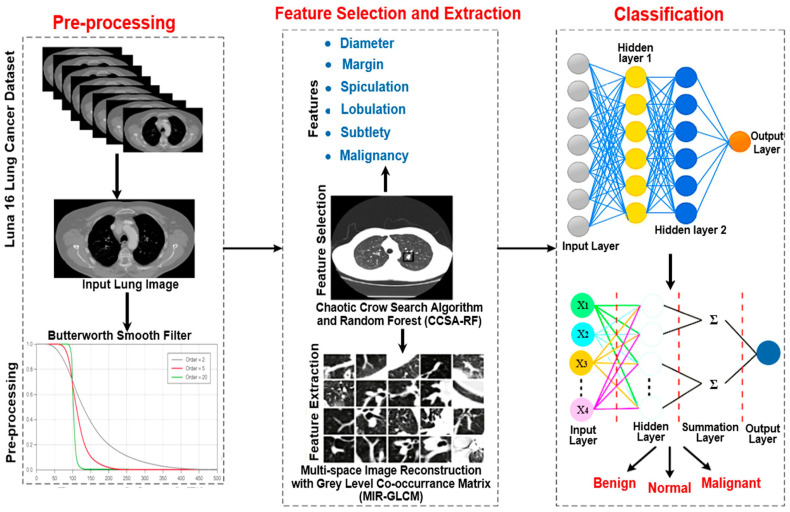
The overall architecture of the proposed algorithm for lung tumor detection.

This research proposed an algorithm for lung cancer detection from 3D CT images using a deep learning approach. The detection accuracy is enhanced by performing several processes, such as pre-processing, feature selection, feature extraction, and classification. Figure 1 shows the overall architecture of the proposed algorithm, which consists of four consecutive processes:

- Smooth filter-based pre-processing;
- Bi-level-based feature selection;
- Feature extraction;
- Severity classification of the lung tumor.

#### 2.2.1. Smooth Filter-Based Pre-Processing

In this research work, the lung CT images were acquired from the LUNA 16 lung cancer dataset and pre-processed using the Butterworth high-pass filter (BHPF), which has the functionality of significantly removing the noise at low frequency for the following feature extraction phase, but at the same time smoothening the entire CT image, while preserving the resolution. This filtering process involves several key steps (Figure 1): firstly, NumPy and SciPy libraries for mathematical operations and signal processing are imported. Secondly, the lung cancer dataset obtained from LUNA 16 is loaded, and finally, the Butterworth filter is applied to the loaded data. The filtering process involves defining the parameters, such as order and cut-off frequency; the first determines how quickly the filter attenuates the high-frequency components, while the cut-off frequency is the point beyond which filtering occurs [42].

Both parameters affect the image’s sharpness. If the order of the Butterworth High-Pass filter (BHPF) increases, the transition from the pass- to the stop-band is abrupt, and a ringing effect occurs. In contrast, to reduce the ringing effects, the Butterworth high-pass filter (BHPF) attenuates values below the cut-off frequency and presents the transition for values greater than it; if the BHPF order is relatively low and the cut-off frequency is increased, the filter is smoother and exhibits less distortion. Thus, the Butterworth filter’s progressive transition effectively reduces noise while maintaining image quality. The convolution function Μ(Υ, Κ) of a BLPF is expressed as follows:

(1) $$Μ\left(\Upsilon ,\;Κ\right)=\frac{1}{\left(1+{\left(\frac{d(\Upsilon ,\;Κ)}{d_{i}}\right)}^{2n}\right)}$$

whereas the convolution function of a BHPF is expressed as follows:

(2) $$Μ\left(\Upsilon ,\;Κ\right)=\frac{1}{\left(1+{\left(\frac{d_{i}}{d(\Upsilon ,\;Κ)}\right)}^{2n}\right)}$$

where, n represents the order of the filter, d(Υ, Κ) represents the distance measured from the origin, and $d_{i}$ is the frequency at which the filter is switched off.

The different steps implemented in the CT image pre-processing are the following:

- Step 1: provide the input image;
- Step 2: Convert the input image to an array;
- Step 3: Use Fast Fourier Transform (FFT) to change the Fourier frequency of the resulting array;
- Step 4: Use Equation (1) to calculate the convolution function for the smooth filter;
- Step 5: Arrange the convolution function provided in Step 4 with the array created in Step 3;
- Step 6: Inverse FFT is used to calculate the magnitude;
- Step 7: Transform the resultant array to an image;
- Step 8: Perform all the procedures for the BHPF; to create the convolution function for the BHPF, Equation (2) is used instead of Equation (1);
- Step 9: Calculate the PSNR (Peak Signal-to-Noise Ratio) value.

**Figure 2:**
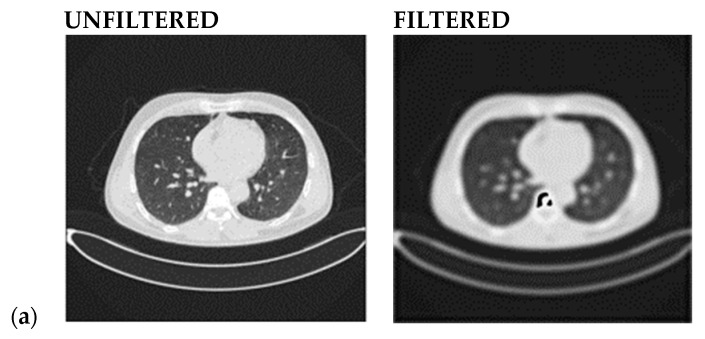

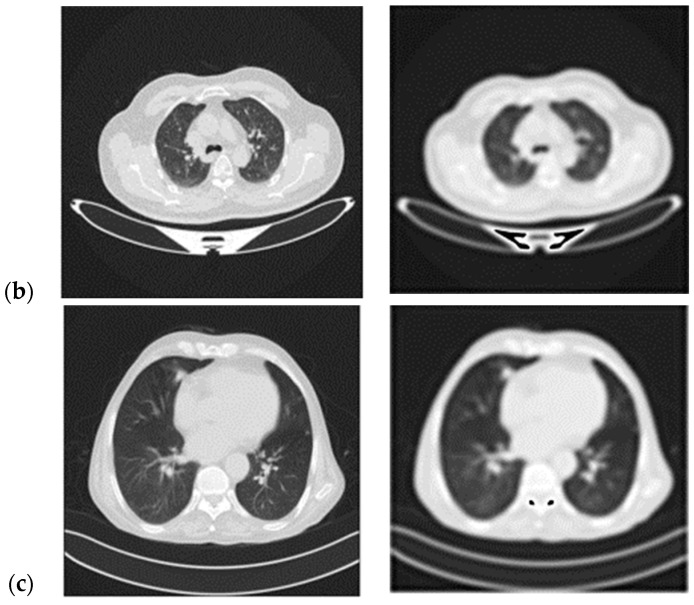
Unprocessed (input) and filtered (output) images obtained by the Butterworth high-pass smooth filter: for a normal case (**a**), for a benign lung-cancer pathology (**b**), and in the case of a malignant lung cancer (**c**).

After defining the filter function, it is applied to the lung image data. The results can be evaluated by comparing the original and smoothed images to determine the smoothing process’s effectiveness. Finally, the smoothed data are saved for the subsequent feature selection process. Figure 2 compares the original image (input) and the filtered one (output) for normal, benign, and malignant lung cancer. It is important to report that the smooth high-pass Butterworth filter, capable of eliminating noise from the image background, produced minimal blurring due to reduced low-frequency noise, but the resolution is preserved. This functionality of the BHPF, expected because it operates differently than Gaussian or Gabor filters, makes it very suitable for the Chaotic Crow algorithm, used to select the best appropriate features that subsequently fed the Random Forest (RF) classifier. In conclusion, the Butterworth filter can be a valuable pre-processing step for feature extraction, especially when dealing with noisy images.

#### 2.2.2. Bi-Level-Based Feature Selection

After the Butterworth smooth filtering process, the next step involves an integrated approach, namely the Chaotic Crow Search Algorithm and Random Forest (CCSA-RF), for feature selection in medical image analysis. This approach focuses on crucial features such as diameter, margin, spiculation, lobulation, subtlety, and malignancy, contributing to a more accurate and efficient analysis of medical images, particularly in the context of a lung cancer diagnosis. The CCSA is a metaheuristic optimization algorithm inspired by the hunting behavior of crows, incorporating chaotic dynamics to enhance exploration and exploitation capabilities. The algorithm iteratively refines the feature subset by adapting to the dataset’s characteristics. This adaptive nature makes CCSA well-suited for optimizing the selection of relevant features in medical image analysis, such as diameter, margin, and malignancy. In this section, the random variables used to update the row position are replaced with chaotic variables. A chaotic sequence formed from chaotic maps is employed since updating the crowd position affects the optimum solution and convergence rate. Ten distinct chaotic maps are employed in this optimization procedure.

These maps may considerably increase the convergence rate and CCSA performance. Equation (3) describes the CCSA technique in conjunction with chaotic sequences.

(3) $$\alpha^{l,\tau +1\;}=\left\{\begin{array}{c} \alpha^{l,\tau \;}+\varsigma_{l}\times {\kappa i}^{l,\tau }\times \left({Ν}^{y,\tau -}\alpha^{l,\tau \;}\right),\;\\ Choose\;a\;r\;and\;position, \end{array}\right.$$

where $\alpha^{l,\tau +1\;}$ is the updated position of the crow, *Ki* is the flight length, in which the high values contribute to the global search and low values to the local search; ς(y) is the chaotic map’s acquired value at the $y_{th}$ iteration, and ς(l) is the chaotic map’s obtained value at the $l_{th}$ iteration. In this research, a new binary CCSA for feature selection is proposed, with the solution pools limited to {0, 1}. The following equations are used to transfer from continuous to binary space.

(4) $$\alpha^{l,\tau +1\;}=\left\{\begin{array}{c} {1,\;\;if\;(v(\alpha }^{l,\tau +1\;}))\geq rand\;(),\;\\ 0,\;\;\;\;\;\;\;\;\;Otherwise\;\;\;\;\;\;\;\; \end{array}\right.$$

where

(5) $$v(\alpha^{l,\tau +1\;})=\frac{1}{1+e^{10{(\alpha }^{l,\tau +1\;}-0.5)}}$$

where the updated binary location at iteration τ is represented by $\alpha^{l,\tau +1}$, and “*rand* ()” is a random integer from a uniform distribution [0, 1]. This study uses CCSA as a wrapper approach based on a feature selection algorithm. A chaotic sequence is included in CCSA’s search iterations. Using CCSA, the best feature subset that describes the dataset is chosen. Feature selection aims to decrease computing costs, shorten feature subsets, and enhance the classification performance.

- Parameter Initialization

Initially, the CCSA process begins with establishing movable parameters and randomizing crow placements (solution) in the search space. With a varied number of features and different lengths, each location indicates a feature subset distinct from the others. CCSA’s original parameter settings are reported in Table 2.

- Fitness Function

Each iteration evaluates the location of each row by a given fitness function. Using m-fold techniques, the data are randomly split into two distinct parts: training and testing datasets. To ensure the stability of the observed results, h is set to 10 in this investigation. The proposed algorithm employs two objective criteria for assessment, namely the classification accuracy and quantity of chosen features. The accepted fitness function equation sets a weight factor as in Equation (6) to integrate the two criteria into one. The classification accuracy is determined by dividing the total number of occurrences by the number of properly classified instances.

(6) $$F_{\tau }=max\left(Accuracy+{ℷ}_{F}\times \left(1-\frac{L_{F}}{L_{\tau }}\right)\right)$$

where *Accuracy* is the classification accuracy, $L_{F}$ is the number of the selected features, $L_{\tau }$ is the number of total features, and ${ℷ}_{F}$ is the weighted factor with a value in [0, 1].

- Position Updating

The update of the crow positions of CCSA is obtained using Equations (4) and (5).

- Termination Criteria

When the maximum number of iterations has been reached, or the optimal solution discovered, the optimization process reaches its conclusion. In this work, we used as many iterations as possible; in each test, the maximum number of iterations that can be performed was set to fifty. A CCSA pseudo-code representation is reported in Algorithm 1.
**Algorithm 1: Chaotic crow search algorithm**Initialize the crow position α at random Calculate the fitness function F(α) for each crow Start the search by initializing the crow Q’s memory // Initialization of the counter Set τ: = 1 Repeat    For l = 1 to H do        Get the value of Chaotic map ς        If ς_x ≥ AP^(x,τ) then            α^(l,τ+1) = α^(l,τ) + ς_l × L_F × (Q^(x,τ) − α^(l,τ))        Else            α^(l,τ+1) = A random position in the search space        End if // Ensure viability of the new position        If (v(α^(l,τ+1)) ≥ r) then            α^(l,τ+1) = 1        Else            α^(l,τ+1) = 0        End if    End for    Verify the viability of α^(l,τ+1)    Analyze the crow’s new location F(α^(l,τ+1))    Update the crow’s memory using Q^(l,τ+1)    Set τ = τ + 1 Until (τ < τ_max_) // Produce the best solution Q

- Random Forest classifier

After the feature selection process facilitated by CCSA, the chosen features are fed into a Random Forest (RF) classifier. In this integrated approach, Random Forest serves as a robust tool not only for classification but also for feature analysis. The Random Forest’s ensemble learning methodology, which combines multiple decision trees, is leveraged to gain insights into the importance and contribution of each selected feature.

The efficacy of the RF classifier is evaluated using the subsequent procedures:

- Step 1: a random forest is a collection of decision tree algorithms used together as an ensemble. This technique extends the bootstrap aggregation (bagging) concept applied to decision trees, making it suitable for classification and regression tasks. Multiple decision trees are generated in bagging, each built from a distinct bootstrap sample of the training data.

The training dataset can be calculated by:

(7) $$U=\{\left(Y_{j},X_{j}{)}_{j=1}^{N}|X_{j}\in \gamma^{M},X\in \left\{1,2,\ldots c\right\}\right\}$$

- Step 2: once the random forest trees and classifiers have been built (Equation (7)), the predictions may be formed by running the test data through the rules of each decision tree to forecast the outcome, which is then stored.

In Equation (7), $Y_{j}$ represent the predictor features [43]; an RF model is created in Algorithm 2, where X is the class responder function, N is the number of training samples, and M is the set of features.
**Algorithm 2: Developed RF model**Input: Dataset {(Y_j, X_j)|j = 1 to N, X_j ∈ γ^M, X ∈ {1, 2, …, c}} Output: Random Forest classifier For k = 1 to K do:    Create bagged subset U_k from the whole dataset U While stopping criteria are not satisfied do:        Select randomly *mtry* features        For each feature in *mtry* do         Calculate reduction in node impurity         Select the feature that reduces impurity the most         Split the nodes into two child nodes End For Construct the K trees to form the RF classifier

- Step 3: collect the votes from several decision trees and decide the test object’s final classification. In other words, the final prediction made by the ensemble model is presented by Equation (8) [43], which defines the k trees of the developed RF classifier:
  (8) $$\hat{X}=Majority\;Vote\;\{\hat{X^{k}}{\}}_{1}^{K}$$
  where $\hat{X^{k}}$ is the predicted outcome of the *k^th^* individual model (in this case, a decision tree $\tau_{k}$) within the ensemble (*K* is the total number of models in the ensemble).

- Step 4: the prediction errors allow for the adjustment and validation of the random forest classifier during training. To achieve this goal, one can utilize the technique of Bagging, which involves training the model using random sub-sets of the training data in order to reduce the variance. Additionally, the Out-of-bag (OOB) error estimate can be calculated by averaging the errors obtained from predictions made by the trees that were not trained on their respective bootstrap samples (Equation (9)). This allows for the random forest classifier to be fine-tuned and verified during the training process [43].

Noteworthy, each tree is built from a bagged sample set, only two-thirds of the samples in U, or “in-bag samples”, are used to construct each tree. The so-called out-of-bag (OOB) samples, representing approximately one-third of the missing data, are utilized to compute the prediction errors. The OOB’s estimated value is as follows:

(9) $${\hat{X}}^{O}=(1/|\left|O_{j^{\prime }}\right||)\sum_{k\in O_{j^{\prime }}}{\hat{X}}^{k}$$

where $\left|\left|O_{j^{\prime }}\right|\right|$ is the size of OOB sub-data, calculated as follows:

(10) $$O_{j^{\prime }}=U\backslash O_{j^{\prime }},j\;and\;j\prime$$

So, the error of prediction in OOB is calculated as follows:

(11) $${\hat{Error}}^{OOB}=\frac{1}{N_{O}}\sum_{j=1}^{N_{OOB}}\zeta (X,{\hat{X}}^{O})$$

where ζ(.) is the “error function” and $N_{O}$ the OOB sample size. To assess this relevance, the difference in mean error between the original and randomly adjusted mean errors in out-of-band data is considered [43]. The technique employs the RF model to anticipate this permuted feature and achieve an error by randomly rearranging all the values of the *j*_th_ feature in OOB before each tree. This permutation aims to examine the impact on the RF model by removing the current correlation between the i_th_ feature and X values. If there is a significant decrease in the mean error, the feature is said to be in strong association; if the RF is increasing, other measures of significant features can be considered. Indeed, once a decision tree has been built, the split is decided at each node *t* by the reduction of the node’s impurity. If a sub-data in node *t* includes samples from c classes, the Gini(t) function is defined as follows:

(12) $$\gamma \left(t\right)=1-\sum_{i=1}^{c}{\hat{P}}_{i}^{2}$$

where ${\hat{P}}_{i}^{2}$ represents the relative frequency of class I, namely the probability of choosing an item with label *i* in the given period. If the classes in t exhibit skewness, the Gini(t) value will be reduced (∆γ(t) represents the Gini index). Once *t* is split into two child nodes, $t_{1}$ and $t_{2}$ with sampling sizes $N_{1}(t)$ and $N_{2}(t)$ respectively, normalized by N(t) that represents the total number of samples at node *t*, the Gini index of the resulting data is determined in the following manner [43]:

(13) $$G_{\delta }\left(t\right)=\frac{N_{1}\left(t\right)}{N\left(t\right)}G\left(t_{1}\right)+\frac{N_{2}\left(t\right)}{N\left(t\right)}G\left(t_{2}\right)$$

To split the node, the feature that offers the least $G_{\delta }\left(t\right)$ is selected. Within a single decision tree $T_{k}$ the relevance score of features $Y_{i}$ is evaluated as follows:

(14) $$\omega_{k}\left(Y_{i}\right)=\sum_{t\in T_{k}}∆\gamma \left(t\right),$$

It is computed over all k trees in an RF, defined as follows:

(15) $$\omega \left(Y_{i}\right)=\frac{1}{k}\sum_{k=1}^{k}\omega_{k}\left(Y_{i}\right)$$

It is essential to remember that a random forest model creates an in-bag significance score by using samples from in-bags, which is the leftover difference between an in-bag importance value and an out-of-bag measure, produced when the prediction error in OOB samples using RF diminishes. Therefore, it takes less time to calculate the in-bag relevance value than the out-of-bag score. Let D be the total quantity of characteristics that predict, which indicates the number of noisy features. Then, let (M−D) be the relevant characteristics that strongly correlate with X values. The total number of potentially uninformative ${∁}_{M-D}^{m}$ and the total number of fall subset features is ${∁}_{M}^{m}$ if we utilize simple random sampling while constructing trees to choose a subset of *m* features (m≪M).The likelihood of selecting a subset of m(m>1) significant characteristics is provided by the following:

(16) $$\frac{{∁}_{M-D}^{m}}{{∁}_{M}^{m}}=\frac{\left(M-D\right)\left(M-D-1\right)\ldots \left(M-D-m+1\right)}{M\left(M-1\right)\ldots (M-m+1)}$$

(17) $$=\frac{\left(1-\frac{D}{M}\right)\ldots \;1-\frac{D\;}{M}-\frac{m}{M}+\frac{1}{M}}{\left(1-\frac{1}{M}\right)\ldots \left(1-\frac{m}{M}+\frac{1}{M}\right)}⋍{\left(1-\frac{D}{M}\right)}^{m}$$

The probability in (17) reduces to 0 because the percentage of significant features is so tiny, indicating that the key features are rarely chosen using the straightforward sampling procedure in RF. First, using all *p*-values for all attributes, we chose an acceptable level of significance as the threshold *θ*, for instance, θ = 0.05. Features whose value exceeds *θ* were deemed uninformative and were eliminated from the system; in the absence of such features, the association with X was evaluated. Afterward, the features Y acquired from H after excluding all non-informative features can be examined. A correlation function $Y^{2}(\tilde{Y},\;X)$ was obtained to examine the link between the categorical response characteristics and each feature $Y_{i}$. Secondly, the optimal subset of features associated with the response feature was selected. Based on the values of $\left(\tilde{Y},\;X\right)$, each observation was assigned to one cell in a two-dimensional array of cells (referred to as a contingency table). When the number of total samples is *N*, and the table has Γ rows and ξ columns, the test statistic value is as follows:

(18) $$Y^{2}=\sum_{j=1}^{r}\sum_{i=1}^{∁}\frac{{\left(O_{j,i}-{Ε}_{i,j}\right)}^{2}}{{Ε}_{i,j}}$$

The main stages included in the proposed algorithm are (i) assign weight to the features by using the feature permutation technique and (ii) determine those features are not biassed and divide them into two groups identified as $Y_{s}$ and $Y_{\varphi }$. (iii) construct RF using the subspaces that include randomly and independently extracted features from $Y_{s}$ and $Y_{\varphi }$, and (iv) categorize the new data set. Below is a synopsis of the developed algorithm:

- By altering the matching predictor feature values for shadow characteristics, create the extended dataset $s_{Y,v}$ of two dimensions;
- Construct an RF model from $\left\{s_{Y,v},\;X\right\}$ and calculate *R* replicates of raw importance scores to use RF for predictor features and shadows. To build the comparison sample $\omega_{v}^{max}$ elements, extract the greatest significance score of each duplicate;
- To determine the weight of each predictor feature, take the *R* importance values and calculate the *p*-value using the Wilcoxon test;
- Ignore uninformative characteristics if there is a significance level threshold θ;
- Divide the remaining features into two subsets, $Y_{s}$ and $Y_{\varphi }$ (as in Algorithm 3);
- Sample the replacement-to-generate-bagged samples $H_{1},{\;H}_{2},\ldots ,\;H_{k}$ from the training set H;
- For each $H_{k},$ grow a CART tree $T_{K}$ as follows:
- For every node, choose a subspace of mtry (mtry>1) features at random and apart from $Y_{s}$ and $Y_{\varphi }$. Then, utilize the subspace features as candidates to divide each node;
- Every tree is grown non-deterministically, without pruning, until the minimum node size $n_{min}$ is reached;
- Given a $Y=y_{new}$, use step (1) to predict the response value.

This step allows for a deeper understanding of how features such as diameter, margin, and malignancy influence the overall analysis and classification of medical images.
**Algorithm 3: Random Forest**Input: H and an RF training dataset: number of replicates and threshold. Output: $Y_{s}$ and $Y_{\varphi }$    Let $s_{Y}=\left\{\frac{H}{X}\right\},M=||s_{Y}||$    For r ←1 to R do        $s_{v}\leftarrow permute\left(s_{Y}\right)$        $s_{Yv}=s_{Y}\cup s_{v}$       Build RF model from $s_{Yv}$ to produce $\left\{\omega_{Y_{i}}^{\gamma }\right\}$       $\left\{\omega_{v_{i}}^{\gamma }\right\}$ and $\omega_{v}^{max},\;\left(i=1,\ldots ,\;M\right).$   Set $\tilde{Y}=\theta$   For i ←1 to M do        Compute the Wilcoxon rank- sum test with $\omega_{Y_{i}}$ and $\omega_{v}^{max}$        Compute $p_{i}$ values for each feature $Y_{i}$       if $p_{i}\leq \theta$ then            $\tilde{Y}-\tilde{Y\;}\cup Y_{i}\left(Y_{i}\in s_{Y}\right)$   Set $Y_{s}=\theta ,Y_{w}=\theta \;$   Compute $Y^{2}(\tilde{Y,}X)$ statistic to get $p_{i}$ value   for i←1 to $\left||\tilde{Y}|\right|$ do      if $\left(p_{i}<0.05\right)$ then            $Y_{s}=Y_{s}\cup Y_{i}\left(Y_{i}\in \tilde{Y}\right)$   $Y_{w}=\{\tilde{Y}\backslash Y_{s}\}$    Return    $Y_{s},Y_{w}$


#### 2.2.3. Feature Extraction

Multi-space Image Reconstruction with Grey Level Co-occurrence Matrix (MIR-GLCM) is used to increase the accuracy of the analysis. GLCM is a texture analysis technique that characterizes the spatial relationships between pixel intensities in an image. The MIR approach shares information from various spatial scales, providing a more comprehensive understanding of the image structure. The MIR technology is a feature extraction method enabling accurate and clear image reconstruction, leading to classification accuracy greater than 99.5%. Compared to the previous average of 10 frames per second, the processing time has been lowered to an average of 12 frames per second. In the following equations, ∂ represents the enhanced modified optimization function, ϑ the modified reconstruction function, $\tau_{s}$ is a weight constant, and s is a sparsity penalty.

(19) $$\partial =\vartheta +\tau_{s}s$$

where

(20) $$\vartheta =\sum_{j}^{N}(\alpha_{j-1}^{out}-\alpha_{j}^{in}{)}^{2}-\varphi$$

(21) $$S=1^{n}\sum_{i=1}^{M}(-r_{i}^{\epsilon }\log r_{i}^{\epsilon })$$

Recreating the image with the MIR aid is easier, providing a clear reconstructed image, thus making it easy to classify cancer from the reconstructed image. To provide a more accurate image reconstruction, the MIR technique reconstructs the image into its many components and then combines them. Increasing the filter window’s size can reduce the time required for processing since it enables more data to be processed simultaneously. Incorporating texture features through GLCM enriches the feature set, contributing to a more detailed analysis of medical images. Therefore, including MIR with GLCM enhances the overall accuracy, making it a promising method for medical image analysis in the context of lung cancer detection.

In particular, the feature extraction step allows you to emphasize and decrease the image dimensionality. Feature extraction is a quantitative data selection method from easily accessible characteristics for classifying object types and properties of each pixel. Item recognition necessitates using parameters that define the item in question; the shape, color, size, and texture are examples of these attributes. Each item is extracted for its features and assigned to a class based on the specified characteristics. The GLCM feature extraction technique provides a matrix that defines the probability of two pixels with different brightness occurring at different distances and angles within an image. GLCM feature extraction is performed in four angular directions, each with a 45° interval: 0°, 45°, 90°, and 135°. The texture analysis extracts the grayscale properties of an object that distinguish it from other objects; contrast, correlation, energy, and homogeneity are among the extracted features.

- Contrast

The degree of difference in the grayness of an image is calculated using the contrast characteristic; it is higher when there is a greater difference in the greyness of two colors. The contrast, however, will decrease proportionally to the degree to which the difference in greyness between two pixels is less significant. The contrast definition is as follows:

(22) $$C=\sum_{j}\sum_{i}(j-i{)}^{2}p(j,i)$$

where *C* is the contrast and p(j,i) is the generic element of the GLCM matrix.

- Correlation

Correlation determines the connection of a reference pixel with its neighbors throughout the image; it is defined as follows:

(23) $$C_{r}=\sum_{j}\sum_{i}\frac{ji\rho_{d}\left(j,i\right)-\mu_{\alpha }\mu_{\beta }}{\sigma_{\alpha }\sigma_{\beta }}$$

where the mean and standard deviation of the probability matrix GLCM along row-wise α and column-wise β are denoted by the $\mu_{\alpha }\mu_{\beta }$ and $\sigma_{\alpha }\sigma_{\beta }$, respectively.

- Energy

The energy value of an image represents the degree of gray distribution in the image. It is defined as follows:

(24) $$E=\sum_{j}\sum_{i}p^{2}(j,i)$$

where *p*(*j*,*i*) is the normalized value of the gray-scale at positions j and i of the kernel with a sum equal to 1.

- Homogeneity

The degree of greyness homogeneity is statistically calculated from the standard deviation of each pixel from the mean gray value. The homogeneity value is higher in images with a practically identical degree of greyness. The homogeneity is defined as follows:

(25) $$H=\sum_{j}\sum_{i}\frac{p(j,i)}{1+|j-i|}$$

#### 2.2.4. Lung Tumor Severity Classification

For classification purposes, the proposed Sparse Convolutional Neural Network with Probabilistic Neural Network (SCNN-PNN) is used to refine the analysis and enhance the accuracy of lung cancer detection. This hybrid method combines the strengths of these two techniques to reconstruct high-resolution CT images and improve the precision of cancer detection. The first component of the proposed method involves the use of CNNs, well-known for their ability to learn hierarchical features from data, and the sparse variant introduces sparsity constraints to enhance the feature learning efficiency. In this context, the Sparse CNN is utilized to reconstruct predicted high-resolution patches, with the aim to enhance the resolution of the CT images, providing a more detailed representation of lung structures.

- Sparse Convolutional Neural Network (SCNN)

Suppose we have a series of pairs of low- and high-resolution training CT images. For each pair, the high-resolution image is used as the mapping target of the low-resolution image. A deep SCNN is used to represent the mapping; it receives the low-resolution picture as input and produces the high-resolution image as output. During the training phase, bicubic interpolation was employed to increase the image resolution. Following this, we proceed to extract features from the up-scaled picture. During image restoration, a common method for feature extraction is first to densely extract the patches and then represent them using a collection of pre-trained bases, such as MIR-GLCM. Thus, the input up-scaled low-resolution image $I_{low}$ first goes through a convolutional layer:

(26) $$H_{1}\left(I_{low}\right)=max(0,F_{1}\times I_{low}+\beta_{1})$$

To extract features for each patch, where F1 denotes the filters, $\beta_{1}$ the biases, and × the convolution operation. To construct the accurate mapping from low to high resolution, the obtained sparse vector is mapped to the high-resolution sparse coefficient by a non-linear mapping $H_{3}$ based on the restricted linear unit (ReLU) method. Then, a high-resolution dictionary $D_{High}$ was used to reconstruct the dense features of high resolution from sparse coefficients and then feed the features into the final layer $H_{4}$ to reconstruct the high-resolution patches. The expected overlap patches are averaged to create the final full image. This process can be compared to applying a customized filter to a set of feature maps, where each point is the “flattened” vector version of a high-resolution patch. Therefore, a convolutional layer was deployed to produce the final, high-resolution image.

(27) $$H_{4}(\tilde{H}\left(I_{low}\right))=max(0,F_{4}\times H\left(I_{low}\right)+\beta_{4})$$

where H denotes the previous operations, $F_{4}$ is composed of several filters and $\beta_{4}$ is a bias vector. The proposed sparse operator $H_{2}$ is based on an intimate connection between sparse coding and neural networks. After feature extraction, for a given input vector:

(28) $$Χ=H_{1}\left(I_{low}\right)\;\epsilon \;R^{n}$$

With a given low-resolution dictionary $D_{low}$, the objective is to find the most efficient sparse code combination; the vector $\partial \;\epsilon \;R^{m}$, which minimizes an energy function that combines the square reconstruction error and a $l_{1}$ sparsity penalty on the code, is the source of the energy function.

(29) $$arg\underset{\partial }{\min}\frac{1}{2}|\left|Χ-D_{{low}^{\partial }}\right|{|}_{2}^{2}+\propto |\left|\partial \right|{|}_{1}$$

The variable ∝ is a coefficient that controls the sparsity penalty and $D_{low}$ is a dictionary matrix with dimensions of n×m. The columns of this matrix contain the normalized basis vectors. To adaptively optimize the sparse coefficient, firstly, the low-resolution dictionary is initialized by randomly set $D_{low}(0)$ with Gaussian noise. Then, the dictionary and sparse code are iteratively optimized:

(30) $$\partial_{k+1}=h_{\theta }\frac{1}{L}D_{low}\left(k\right)x+(1-\frac{1}{L}{D_{low}}^{T}\left(k\right)D_{low}\left(k\right))\partial_{k}$$

(31) $$D_{low}\left(k+1\right)=\begin{array}{c} \arg\\ D_{\mathrm{low}} \end{array}min\frac{1}{2}|\left|x-D_{low}\left(k\right)x\right|{|}_{2}^{2}+\propto |\left|\partial_{k+1}\right|{|}_{1}$$

where $h_{\theta }$ denotes the shrinkage function

(32) $$h_{\theta }\left(v\right){|}_{j}=sign\left(v_{j}\right)\left(\left|v_{j}\right|\right)-\theta_{j}),\theta_{j}=\propto /L\;$$

L is the upper bound on the largest eigenvalue of ${D_{low}}^{T}\left(k\right)D_{low}\left(k\right)$.

- Restricted Linear Unit

Previous layers extract a $n_{1}$-dimensional sparse representation for each low-resolution patch. Learning the connection of low-resolution sparse representations with high-resolution sparse codes is necessary to enhance the NN’s performance. In other words, obtaining an informative and relevant representation that can be highly connected to the high-resolution patch from previous low-resolution representation is needed. We performed a non-linear mapping to build the $n_{1}$-dimensional high-resolution vectors from $n_{1}$-dimensional. This task is comparable to applying filters with a spatial support of 1 × 1, which is considered trivial.

(33) $$H_{3}\left(\partial \right)=max(0,F_{3}\times x+\beta_{3})$$

where $F_{2}$ is comprised of $n_{2}$ filters with dimensions of $n_{1}\times 1\times 1$, and $\beta_{3}$ is a $n_{2}$ dimensional function. In a conceptual sense, each of the output $n_{2}$-dimensional vectors is a sparse representation of a high-resolution patch that will ultimately be used for the reconstruction process. On the filter responses, the Rectified Linear Unit operation (ReLU, max(0, x)) is carried out. To obtain a higher level of non-linearity, it is feasible to include more convolutional layers, which has the potential to increase the complexity of the model ${(n}_{2}\times 1\times 1\times n_{2}$, parameters for one layer), which requires more training time.

- Loss Function

**Figure 3:**
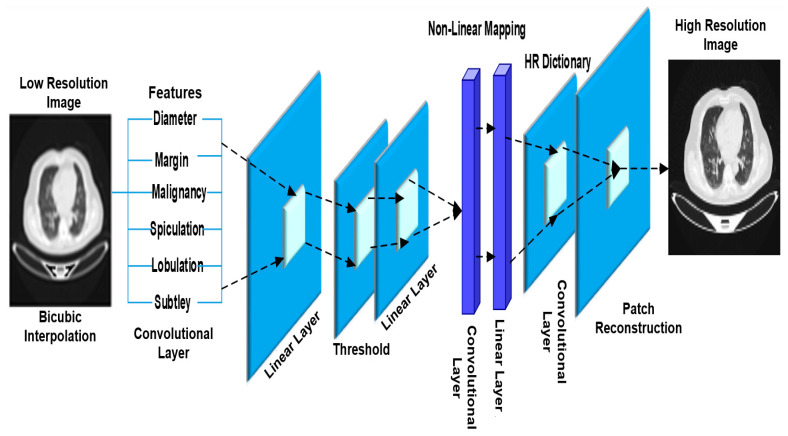
Architecture of the Sparse Convolutional Neural Network.

To become familiar with the function of end-to-end mapping in running SCNN, it is necessary to estimate all network parameters. Figure 3 indicates the SCNN architecture.

(34) $$W={\{F}_{1},\beta_{1},D_{low},\propto {F_{3},\beta }_{3},D_{High},{F_{4},\beta }_{4}\}$$

These parameters are achieved by reducing the amount of loss between the reconstructed images SCNN $\left(I_{low},W\right)$ and corresponding real high-resolution (HR) ones. The mean squared error (MSE) and loss function are used on a collection of high-resolution images denoted by $\left\{{I^{i}}_{High}\right\}$ and the equivalent low-resolution ones denoted by $\left\{{I^{i}}_{Low}\right\}$.

(35) $$L\left(W\right)=\frac{1}{n}\sum_{j=1}^{n}||F_{SCNN}\left(I_{Low,}^{i}w\right)-I_{High}^{i}|{|}_{2}^{2}$$

**Figure 4:**
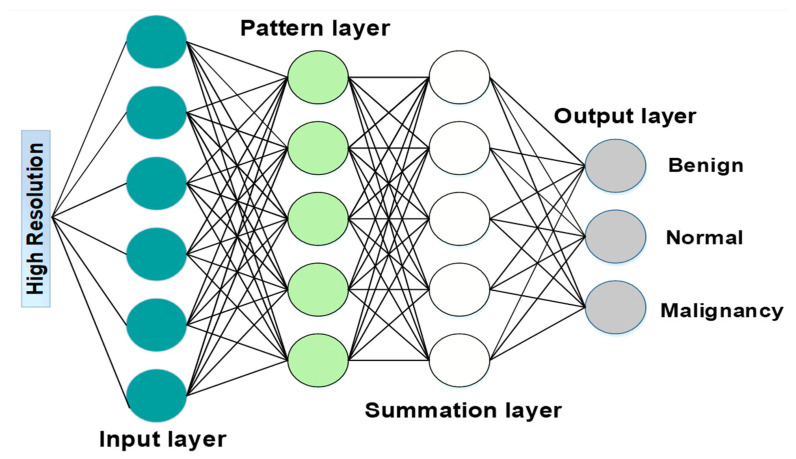
Structure of the developed PNN.

Where n is the total number of training samples used. All parameters of the convolutional layer are set randomly by choosing 0 for bias and 0.001 for standard deviation and mean from a Gaussian distribution. This operation is carried out to ensure that the parameters are accurate. The mean squared error (MSE) of the loss function is determined by calculating the difference between the $\left\{{I^{i}}_{High}\right\}$ and the output of the network. The final high-resolution CT image is obtained by generating the expected high-resolution patches and then averaging them while considering overlapping areas. This reconstruction step contributes to improving the visual fidelity and diagnostic quality of the images, thereby aiding in more accurate lung cancer detection. Simultaneously, a PNN is integrated into the hybrid method for accurate cancer detection. PNNs are well-suited for probabilistic modeling and decision-making tasks and therefore are used to detect cancer with high accuracy. It leverages the information extracted from the feature-selected and classified data to make probabilistic predictions about the presence of lung cancer in the images. A probabilistic neural network (PNN) classifier categorizes pictures to identify aberrant CT images (including pathology) [44]. Probability neural networks (PNNs) were practically indifferent to outliers and provided target probability measures that are properly anticipated in Figure 4. The defined definition of the Bayes rule for the class ∀ is as follows:

(36) $${℘}_{\forall }C_{\forall }f_{\forall }\left(X\right)>{℘}_{b}C_{b}f_{b}\left(X\right)$$

The occurrence of a pattern in class ∀ with a prior probability is denoted by ${℘}_{\forall }$, whereas $C_{\forall }$ is a cost function. Additionally, $f_{\forall }\left(X\right)$ is the probability density function (PDF) for the class ∀ used in this context. The four layers that make up the PNN are the input layer, the pattern layer, the summation layer, and the decision (output) layer.

To distinguish abnormalities from normal CT scans, the following generalized kind of layers were employed throughout the programming process:

- Input: read the input units ∀(℘), ℘ = 1, 2, …, ℘ and connect them to all pattern units.
- Pattern: create a pattern unit $z_{℘}$ (which is either $z_{\forall }$ or $z_{b}$ unit) with weight vector:
  (37) $$w_{℘}=X(℘)$$
- Summation: if X(℘) goes to class ∀, connect pattern unit $z_{℘}$ to the summation unit $S_{\forall }$, otherwise to unit $S_{b}$. The weight used by the summation unit for class B is calculated according to Equation (37), where $m_{\forall }$, is several training patterns in class ∀.
  (38) $$v_{b}=\frac{{℘}_{b}C_{b}\;m_{\forall }}{{℘}_{\forall }\;C_{\forall }\;m_{b}}$$
- Decision: if the entire input to the decision unit is positive, the input vector is categorized as belonging to the class ∀.

The speed at which PNN can be taught is the most significant benefit it offers. In contrast to being “trained”, the weights are allocated. Throughout the training process, only new vectors will be modified or added to weight matrices; established values will never be changed. Given that the manipulation of matrices may carry out the training, the performance of the PNN is excellent. The probabilistic nature of PNNs allows for a nuanced understanding of uncertainty in predictions, contributing to more robust and reliable detection outcomes such as benign, normal, and malignant.

## 3. Results

This section describes the experimental investigations performed to assess the performance of the suggested lung tumor detection method. In addition, this part is divided into three subsections: simulation setup, comparative analysis, and research summary.

### 3.1. Simulation Setup

To simulate the developed lung cancer detection algorithm, Python ver. 3.9.6 is utilized, an efficient tool that provides all specifications for the proposed technique. Table 3 indicates the system specifications.

### 3.2. Comparative Analysis with Other DL Models

This section compares the proposed SCNN-PNN approach to existing ones, such as the DenseNet201 and CNN+ SVM models, evaluating its efficacy using performance metrics such as accuracy, precision, F1-Score, sensitivity, and specificity.

- *Accuracy*

The classification model’s accuracy is determined by how many of its predictions are accurate. It is expressed using the following equation (Equation (39)):

(39) $$H=\frac{\Theta +\varpi }{\Theta +\varpi +℘+Κ}$$

**Figure 5:**
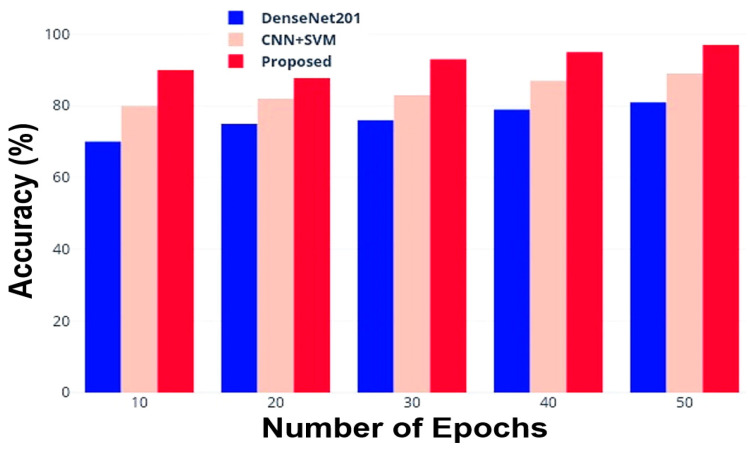
Obtained accuracy for DenseNet201, CNN + SVM, and the proposed model as a function of epochs.

True positive (Θ) represents the number of cancer cases classified as such and appropriately determined. True Negative (ϖ) indicates the number of non-cancer scenarios that were accurately classified as such. False positive (℘) is the number of non-cancer cases incorrectly assigned to cancer classification; instead, false negative (Κ term) is the number of cancer cases incorrectly classified as non-cancerous. Table 4 reports the accuracy outcomes for DenseNet201, CNN + SVM, and the proposed models as a function of the epochs; Figure 5 graphically reports the accuracy results obtained.

- *Precision*

**Figure 6:**
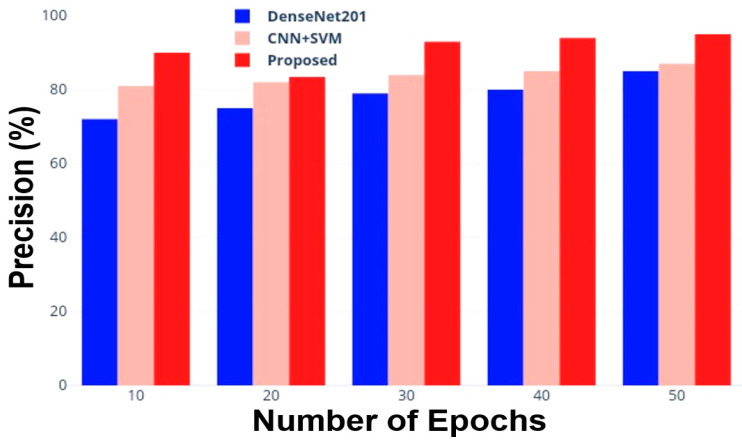
Obtained precision for DenseNet201, CNN + SVM, and the proposed model as a function of epochs.

This metric is the proportion of properly identified positive predictions; Equation (40) is used to determine the precision. The comparative analysis in terms of precision between the DensNet201, CNN + SVM, and the proposed model is represented in Table 5 (as well as in graphic form in Figure 6).

(40) $$Precision=\frac{\Theta }{\Theta +℘}$$

- *F1-Score*

Especially for tasks such as binary classification and image segmentation, the F1 score is a frequently used performance metric in machine learning and image processing. It performs especially well with imbalanced datasets since it integrates recall and precision into a single number. The formula below is used to determine the F1 score (E):

(41) $$E=\frac{2(g\times F)}{g+F}\;$$

where g represents the precision, and F the recall parameter.

**Figure 7:**
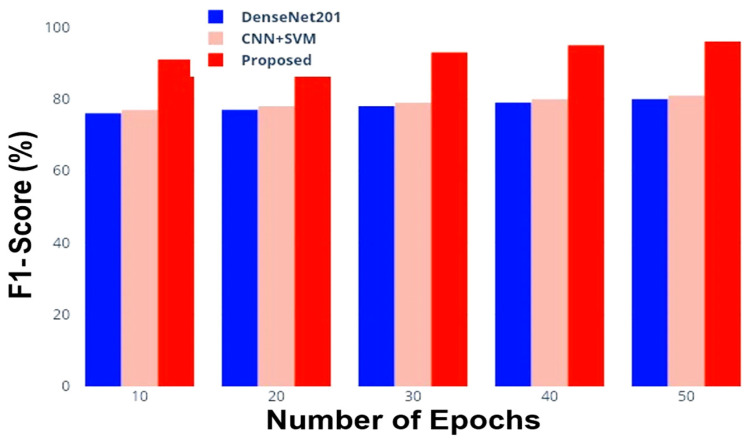
F1-score of DenseNet201, CNN + SVM, and proposed model as a function of epochs.

Figure 7 compares the trends of the F1 score for the considered DenseNet201, CNN + SVM, and the proposed model as a function of the epochs; Table 6 reports the obtained F1 score values for the tested models.

- *Sensitivity and Specificity*

Sensitivity (F) is the fraction of true positives correctly recognized, i.e., the ability to detect individuals affected by the under-test condition. The Equation (42) is used to compute it:

(42) $$F=\frac{\Theta }{\Theta +℘}$$

The proportion of true negatives correctly recognized by the test, namely the test capacity to correctly identify persons who do not have the condition being tested for, is measured by the specificity (b). The following Equation (43) is used to compute it:

(43) $$b=\frac{\varpi }{\varpi +℘}$$

**Figure 8:**
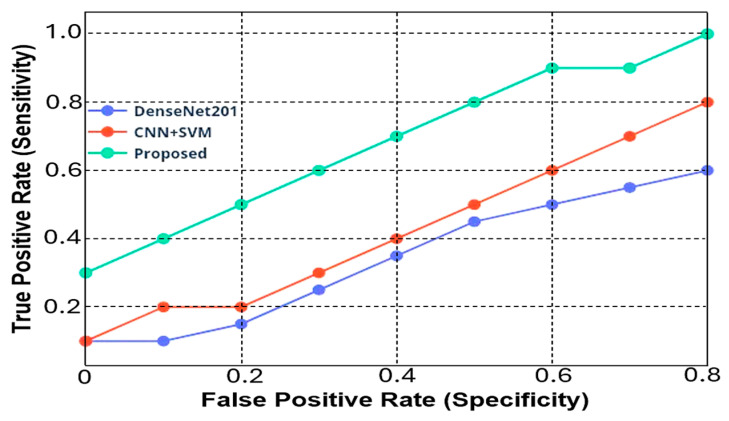
ROC curve of the DensNet201, CNN + SVM, and proposed model as a function of epochs.

Figure 8 reports the sensitivity vs. specificity plots (i.e., the Receiver Operating Characteristic-ROC-curve) for DenseNet201 and CNN + SVM models, and the new approach proposed in this research work. The ROC curve is a fundamental tool for evaluating the trade-off between sensitivity and specificity for binary classification models; it demonstrates how well each model can discriminate between positive and negative classes.

## 4. Results Discussion

This section reports the discussion of the obtained results, presented in the previous section, to extract insights and evaluations about the capabilities of the proposed method compared to other DL methods. As evident from Figure 5, the DenseNet201 reaches an 81.5% accuracy after 50 epochs (iterations); it begins with an accuracy of roughly 70% and steadily improves as the number of epochs grows. The combination of CNN + SVM, on the other hand, starts with a higher accuracy (equal to 80.0%) and similarly shows a constant improvement as the number of iterations increases, finally stabilizing at around 89.0% accuracy. Instead, the developed model demonstrates better performance from the beginning, even with a small number of iterations; in just ten epochs, it achieves a 90.0% accuracy and then reaches the best performance (97.5% accuracy) compared to the other models after 50 epochs. Regarding classification precision (Table 5 and Figure 6), a similar trend is evident, with the proposed approach showing the best performance as a function of the epochs number compared to the tested models already known in the literature. In particular, for a number of 50 iterations, the proposed approach reaches a 95.5% classification precision, whereas the DenseNet201 and CNN + SVM models achieve 85.0% and 87.0% values, respectively. These results demonstrate that the proposed method has better precision during classification than conventional approaches.

Regarding the F1-score values obtained, the DenseNet201 and CNN+ SVM models showed initially low values (equal to 76.0% and 77.0% with 10 epochs, respectively) that gradually improved, reaching 80.5% and 81.5% with the highest number of iterations equal to 50 (as reported in Table 6). Instead, the proposed model provided a significantly higher F1-score ranging from 91.0% with only ten epochs up to 96.5% for a number of iterations equal to 50 (as reported in Table 7). Figure 7 highlights the effectiveness of the proposed model in achieving a higher F1 score compared to existing models. From the specificity and sensitivity point of view, the DenseNet201 model achieved a sensitivity of 0.1 with a specificity of 0, whereas for a specificity of 0.6, it achieved a sensitivity value of 0.50. As regards the CNN + SVM model, at the starting point, it provided a sensitivity value of 0.1, which reached up to 0.8 for a specificity value of 0.8, too. Instead, the proposed hybrid SCNN-PNN method provided a higher initial value of 0.3, which increases up to 0.995 (higher than 0.8 obtained from the other models) for a specificity value of 0.8, thus demonstrating greater efficiency compared to existing methods (Figure 8).

### Performance Comparison with the Scientific Literature

Table 7 compares the results obtained by the proposed SCNN-PNN approach with those of some studies in the literature, such as the Refs. [25,26,27,28,29,30,31,32,33,34,35,36,37,38], already discussed in Section Literature Survey and listed in Table 1. The research works related to Refs. [30,34,35], and [36] were excluded because they were applied to histopathology images of tumor cell biopsies (i.e., not CT lung images). In terms of accuracy, the comparison shows that the SCNN + PNN hybrid model was an appropriate and successful choice for automatic lung cancer diagnosis by CT images analysis. Regarding this consideration, the framework proposed in Ref. [26] features better performance in terms of accuracy (98.7% vs. 97.5%) and precision (98% vs. 95.5%) compared to the model presented in this work, which, however, provides higher sensitivity (99.5% vs. 96.85%). However, it must be considered that the system presented in Ref. [26] presents a greater complexity than the one proposed since it uses a segmentation system of medical images based on the UNETR architecture, followed by a classification section based on a self-supervised neural network.

**Figure 9:**
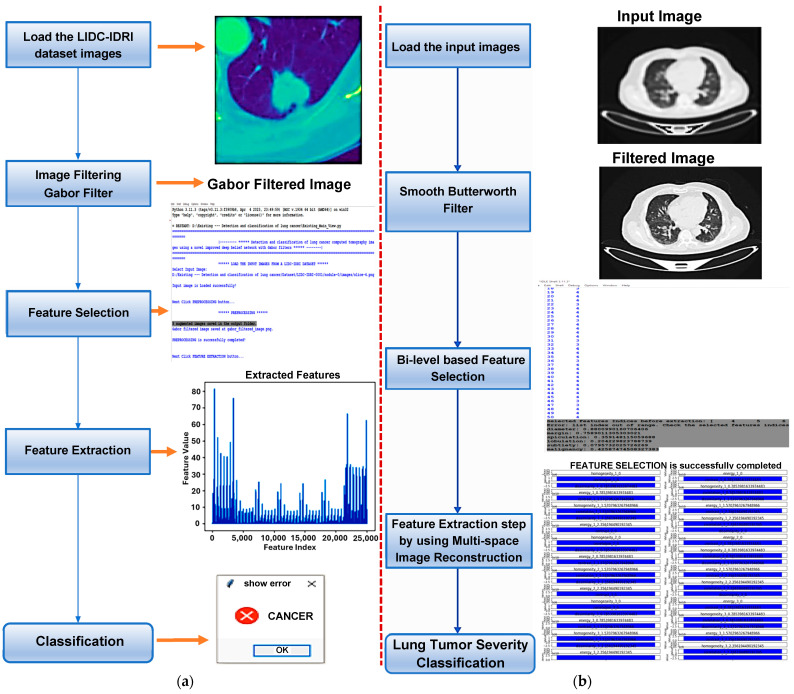
Comparison between the algorithms proposed in Ref. [38] on the left (**a**) and that proposed in this research work on the right (**b**); the dashed vertical line separates the two simplified schemes for clarity.

The studies in Refs. [28,38] are particularly interesting because they were carried out using the LUNA16 dataset, as in this research work. In Ref. [28], the authors did not generate the specificity, which can be attributed to the high rate of false positive responses, as the authors stated, or to the use of the R-CNN-based attention-embedded model, which employs sequential processing of region proposals. The R-CNN model can generate multiple region proposals that overlap significantly, which makes it a resource-demanding approach and relatively slow during inference, affecting the detection performance. Furthermore, R-CNN has separate modules for region proposal and classification, which can lead to suboptimal performance compared to models that optimize both tasks jointly. The technical discussion above may justify the high rate of FP responses in Ref. [28]. The study in Ref. [38] provides better performance than that obtained in this research work; however, the authors in [38] employed Gabor filtering, enhanced Deep Belief Network (DBN), and SVM. The Gabor de-noise filter depends on various parameters that need to be set properly, which makes the filtering process computationally demanding compared to the SCNN + PNN approach developed in this work. Although the DBN, a new DL model, can effectively avoid generating false positive responses, it exhibits highly complex architecture and requires huge amounts of data to perform the classification process. Figure 9 shows a comparison between the model proposed in [38] and that proposed in this article. It is evident that the proposed approach requires much lower computational demand than the DBN. In addition, the framework in Ref [29] reaches a similar performance to the proposed model, using an attention-embedded CNN for pulmonary nodule detection. However, it features limited interpretability and context understanding, high sensitivity to hyper-parameters, and low data efficiency since it requires huge amounts of data for training. 

The remaining scientific works reported in Table 7 were trained and tested on local datasets [26,31,33] or well-known general datasets available on the internet [24,25,27,29]. The results on local datasets may imply a bias in image quality during the study. In contrast, the results derived from general datasets (Table 1) either produced lower performance metrics, such as in Ref. [24], or did not provide the precision metrics, such as in Refs. [25,29], or did not calculate the accuracy, such as in Ref. [27]. Furthermore, these research works provided lower sensitivity than this study. However, they are significant in giving a precise comparison and suggesting testing the proposed SCNN + PNN models on other datasets. To end, if one examines the accuracies reported in all studies in Table 7, it is rational to suggest that the proposed SCNN + PNN model represents another successful CNN choice for automatic lung cancer diagnosis using CT images. 

Regarding the objectives of this research work, Shu et al. designed a balanced distribution active learning (BDAL) framework to select features before the classification phase applied to Magnetic Resonance Imaging (MRI)-based images. They stated that active learning (AL) can successfully select images (or features in this study) to avoid unnecessary calculations, while in this research work, we investigated the SCNN-PNN model [45]. In a second study, the same authors attempted to perform edge detection of targeted objects while resisting the amount of noise inevitably present in medical imaging applications. This study uses Butterworth filtering to eliminate the noise before CNN and classification steps [46], as well as in this manuscript. Both studies [45,46] were performed on medical images relating to pathologies other than CT images and lung nodules, respectively, related to the present research work.

## 5. Conclusions

In conclusion, the proposed AI-based hybrid approach for lung cancer detection successfully exploited the combination of techniques in image pre-processing, feature selection, and classification. The integration of the Butterworth smooth filter for noise reduction and image enhancement, of the Chaotic Crow Search algorithm along with Random Forest for feature selection, and Multi-space Image Reconstruction with Grey Level Co-occurrence Matrix (MIR-GLCM) for feature extraction represented a comprehensive and effective approach for the analysis of lung’s pathological CT images. However, regarding the use of BHPF in the pre-processing of images to be classified, it is crucial to weigh the benefits of noise reduction versus the potential loss of detail based on the features you’re interested in. The tunable parameters (cut-off frequency and filter order) allow you to control the level of smoothing, finding a balance between noise reduction and detail preservation for the following specific features’ selection.

Using a SCNN-based approach with a Probabilistic Neural Network for lung cancer severity classification, with a specific focus on identifying benign, normal, and malignant stages, further improved the precision and efficiency of the diagnostic process. The incorporation of the PNN algorithm helped reduce complexity while ensuring accurate classification results. Evaluation of the proposed methods through performance metrics such as accuracy, precision, F1 score, sensitivity, and specificity provided a robust assessment of their effectiveness. These parameters collectively contributed to an in-depth understanding of the model’s ability to accurately diagnose and classify lung cancer cases. The research has demonstrated the promising potential of artificial intelligence, particularly deep learning, in analyzing pathological images for lung cancer diagnosis. The proposed method addressed the challenges associated with medical image analysis and contributed to the ongoing efforts to enhance the accuracy and efficiency of lung cancer diagnosis and prognosis. As medical imaging technology advances, the integration of sophisticated AI algorithms holds great promise for improving patient outcomes in lung cancer treatment.

The algorithm’s performance on new patients’ data depends on several factors, including the diversity of the training data, consistent pre-processing, images’ quality and resolution, and variations in the device and protocol used to acquire the images. Specifications or parameters of the developed algorithm such as adaptability, validation, handling outliers, appropriate choice of features, and relative flexibility, influence the algorithm’s ability to generalize its performance to new cases/datasets, and adapt to changes in the above factors while maintaining accuracy and relevance unchanged. Based on these considerations, we believe that the developed AI-based hybrid approach for lung cancer detection can maintain the obtained performances unchanged if applied to different datasets or in a real application to newly acquired data/images.

Future work will focus on the comparison of the metrics extracted from malignant tumor CT images with those available in the literature and in various published databases in order to reveal common criteria or parameters among malignant tumors. However, this effort may face limitations, including the diversity of medical imaging devices and pre-processing techniques used, which must be taken into account.

## Figures and Tables

**Table 1:** Summary of the literature survey with the corresponding limitations highlighted.

Work	Objective	The Method or AI Approach	Limitations
A. Sebastian et al. [25]	Detection of Lung Nodules via Enhanced Convolutional Neural Network	Improved Moth Flame Optimization	The model calculation time is high (low computational speed). The model intends to utilize visualization tools to comprehend the networks’ classification components and see whether they match the radiologists’ diagnoses.
Y. Said et al. [26]	Deep Learning-Based Medical Image Segmentation for Lung Cancer Diagnosis.	UNETR network, self-supervised network	The model requires high computational demands and a powerful GPU; this can be overcome by using powerful local machines or cloud platforms.
A. Bhattacharjee et al. [27]	Computed tomography images for the early diagnosis of chronic renal disease and lung cancer.	Xception framework	The Malignant, Normal, and Benign (MNoB) model did not outperform the Normal, Cyst, Tumour, and Stone (NCTS) model despite promising findings due to insufficient training data.
Z. Riaz et al. [28]	Segmentation of Lung Tumour Images from Computer Tomography Images.	MobileNetV2 and UNET	The segmentation was tested only on the challenge validation dataset; to assess its resilience, it has to be tested on other medical image segmentation tasks. The segmentation results were not completely processed.
G. Zhang et al. [29]	Automatic Pulmonary Nodule Detection and Attention-Guided Feature Extraction.	R-CNN-based attention-embedded model, ResNet	A low false-positive rate does not imply sensitivity. Focal loss reduces the information imbalance but cannot eliminate it. Even after data quality improvements, the model needs new data as the network deepens to expand its capability.
G. Kasinathan et al. [30]	Cloud-Based Identification and Stage Categorization of Lung Tumours.	Deep neural model	Cloud system’s memory constraints limit the analysis to no more than 65 records at once, making it inefficient for large hospitals.
S. Mithun et al. [31]	Clinical Concept-Based Radiology Reports Lung Cancer Classification Pipeline.	ML algorithms	The designed pipeline is able to extract data only from HIS but is customizable for other institutions. The rule-based model’s ontology has been customized only using their data. Some false negatives remain unidentified after mapping most of the disease diagnostic keywords.
V. Kumar et al. [33]	Categorization of lung cancer that is malignant.	Median filter, Gaussian filter, Gabor filter, and watershed algorithm.	Overfitting occurs when DL algorithms memorize training data instead of capturing patterns. Overfitting may occur when the model’s capability exceeds the training data. Overfitting can be reduced by data augmentation, dropout, and regularisation, but not eliminated.
M. Lanjewar, et al. [34]	Detecting lung cancer in CT images and feature selection.	DenseNet framework	The quality and representativeness of the training data can influence the model performance. Demographic imbalances or imaging procedure changes can affect model performance and generalizability.
B.R. Pandit et al. [35]	To improve prediction accuracy and save processing time, use multi-space images.	Convolution filter, autoencoder model based on convolutional neural network	Image quality affects MIR’s ability to reconstruct CT images. Image quality issues such as noise, artifacts, and resolution can affect MIR performance and classification accuracy.
Z. Ren et al. [36]	Data-augmented ensemble framework for lung cancer categorization.	Deep Convolutional GAN	LCDAE framework performance can depend on pre-processing, hyper-parameters, and model setups. Small changes in these elements could affect the model’s performance and generalizability.
Z. Ren et al. [37]	To identify early-stage lung cancer.	Deep convolutional GAN, transfer learning model	LCGANT may require a lot of computing resources, limiting its usability in resource-constrained applications.
E. Saddiqui et al. [38]	Lung cancer detection and categorization using computed tomography images.	Deep Belief Network, Gaussian-Bernoulli, support vector machine.	Increased sensitivity for high false positive (FP) rates often follows lower sensitivity for low FP rates.

**Table 2:** Summarizing table with the CCSA process’s parameters.

Variables	Value
H	30
AP	0.1
κi	2
Lower bound	0
Upper bound	1
τMax	50
β	Same as the total number of features in the original dataset

**Table 3:** Specifications of the setup used to simulate the proposed lung cancer detection algorithm.

Software Specifications	Operating System (OS)	Windows 10 (64-bit)
	Tool	Python Ver. 3.9.6
Hardware Specifications	RAM	4 GB
	Hard Disk	500 GB

**Table 4:** Obtained accuracy values for the tested models.

Number of Epochs	Accuracy (%)		
	DenseNet201	CNN + SVM	Proposed Method
10	70.0	80.0	90.0
20	75.5	82.0	91.5
30	76.5	83.5	93.0
40	79.0	87.0	95.5
50	81.5	89.0	97.5

**Table 5:** Obtained precision values for the tested models.

Number of Epochs	Precision (%)		
	DenseNet201	CNN + SVM	Proposed Method
10	72.0	81.0	90.0
20	75.0	82.5	92.0
30	79.0	84.5	93.5
40	80.5	85.5	94.5
50	85.0	87.0	95.5

**Table 6:** Obtained F1-score values for the tested models.

Number of Epochs	F1-Score (%)		
	DenseNet201	CNN + SVM	Proposed Method
10	76.0	77.0	91.0
20	77.0	78.0	92.0
30	78.5	79.5	93.5
40	79.5	80.5	95.0
50	80.5	81.5	96.5

**Table 7:** Performance comparison to example studies in the literature (N.A. = Not Available).

Scientific Work	Dataset	Accuracy [%]	Precision [%]	Sensitivity (Recall) [%]
F. Silva, et al. [24]	LIDC-IDRI	92.5	92.5	92.5
A. E. Sebastian, et al. [25]	Decathlon 2018 Challenge	98.8	N.A.	96.8
Y. Said, et al. [26]	Local	98.3	99.3	98.0
A. Bhattacharjee, et al. [27]	Decathlon 2018 Challenge	N.A.	93.2	86.0
Z. Riaz, et al. [28]	LUNA 16	91.2	N.A.	97.7
G. Zhang, et al. [29]	LIDC-IDRI	97.1	N.A.	95.9
S. Mithun, et al. [31]	Local	91.0	N.A.	94.0
V. Kumar, et al. [33]	Local	Up to 100 (97 Avg.)	Up to 100 (95 Avg.)	N.A.
Z. Ren, et al. [36]	LIDC-IDRI, LUNA16, TCIA	99.4	N.A.	98.5
E.A. Siddiqui, et al. [38]	LUNA 16	99.1%	N.A.	98.0%
Proposed hybrid approach	LUNA16	97.5	95.5	99.5

## Data Availability

Data from our study are available upon request.
